# Dynamic alteration in SULmax predicts early pathological tumor response and short-term prognosis in non-small cell lung cancer treated with neoadjuvant immunochemotherapy

**DOI:** 10.3389/fbioe.2022.1010672

**Published:** 2022-10-06

**Authors:** Taotao Sun, Shujie Huang, Yongluo Jiang, Hui Yuan, Junhan Wu, Chao Liu, Xiaochun Zhang, Yong Tang, Xiaosong Ben, Jiming Tang, Haiyu Zhou, Dongkun Zhang, Liang Xie, Gang Chen, Yumo Zhao, Shuxia Wang, Hao Xu, Guibin Qiao

**Affiliations:** ^1^ Department of Nuclear Medicine and PET/CT-MRI Centre, The First Affiliated Hospital of Jinan University, Guangzhou, China; ^2^ Department of Nuclear Medicine, WeiLun PET Center, Guangdong Provincial People’s Hospital, Guangdong Academy of Medical Sciences, Guangzhou, China; ^3^ Department of Thoracic Surgery, Guangdong Provincial People’s Hospital, Guangdong Academy of Medical Sciences, Guangzhou, China; ^4^ Shantou University Medical College, Shantou, China; ^5^ State Key Laboratory of Oncology in South China, Collaborative Innovation Center for Cancer Medicine, Guangzhou, China; ^6^ Department of Nuclear Medicine, Sun Yat-sen University Cancer Center, Guangzhou, China; ^7^ Department of Pathology, Guangdong Provincial People’s Hospital, Guangdong Academy of Medical Sciences, Guangzhou, China; ^8^ The Second School of Clinical Medicine, Southern Medical University, Guangzhou, China

**Keywords:** non-small cell lung cancer, neoadjuvant immunochemotherapy, major pathological response, iPERCIST-max, 18F-FDG positron emission tomography

## Abstract

**Introduction:** Biomarkers predicting tumor response to neoadjuvant immunochemotherapy in non-small cell lung cancer (NSCLC) are still lacking despite great efforts. We aimed to assess the effectiveness of the immune PET Response Criteria in Solid Tumors *via* SULmax (iPERCIST-max) in predicting tumor response to neoadjuvant immunochemotherapy and short-term survival in locally advanced NSCLC.

**Methods:** In this prospective cohort study, we calculated SULmax, SULpeak, metabolic tumor volume (MTV), total lesion glycolysis (TLG) and their dynamic percentage changes in a training cohort. We then investigated the correlation between alterations in these parameters and pathological tumor responses. Subsequently, iPERCIST-max defined by the proportional changes in the SULmax response (△SULmax%) was constructed and internally validated using a time-dependent receiver operating characteristic (ROC) curve and the area under the curve (AUC) value. A prospective cohort from the Sun Yat-Sen University Cancer Center (SYSUCC) was also included for external validation. The relationship between the iPERCIST-max responsiveness and event-free survival in the training cohort was also investigated.

**Results:** Fifty-five patients with NSCLC were included in this study from May 2019 to December 2021. Significant alterations in post-treatment SULmax (*p* < 0.001), SULpeak (*p* < 0.001), SULmean (*p* < 0.001), MTV (*p* < 0.001), TLG (*p* < 0.001), and tumor size (*p* < 0.001) were observed compared to baseline values. Significant differences in SULpeak, SULmax, and SULmean between major pathological response (mPR) and non-mPR statuses were observed. The optimal cutoff values of the SULmax response rate were −70.0% and −88.0% using the X-tile software. The univariate and multivariate binary logistic regression showed that iPERCIST-max is the only significant key predictor for mPR status [OR = 84.0, 95% confidence interval (CI): 7.84–900.12, *p* < 0.001]. The AUC value for iPERCIST-max was 0.896 (95% CI: 0.776–1.000, *p* < 0.001). Further, external validation showed that the AUC value for iPERCIST-max in the SYSUCC cohort was 0.889 (95% CI: 0.698–1.000, *p* = 0.05). Significantly better event-free survival (EFS) in iPERCIST-max responsive disease (31.5 months, 95% CI 27.9–35.1) than that in iPERCIST-max unresponsive disease (22.2 months, 95% CI: 17.3–27.1 months, *p* = 0.024) was observed.

**Conclusion:** iPERCIST-max could better predict both early pathological tumor response and short-term prognosis of NSCLC treated with neoadjuvant immunochemotherapy than commonly used criteria. Furthermore, large-scale prospective studies are required to confirm the generalizability of our findings.

## Introduction

Non-small cell lung cancer (NSCLC) is the most common cause of cancer-related death worldwide (Siegel et al., 2021), likely because most NSCLC patients (>70%) are diagnosed at advanced stages (Ramos-Esquivel et al., 2017). Immune checkpoint inhibitors (ICIs) have changed the landscape of advanced NSCLC treatment (Ramos-Esquivel et al., 2017; Uprety et al., 2020; Huang et al., 2021; Wang et al., 2021). It has been reported that a combination of ICIs and chemotherapy significantly improve the prognosis of NSCLC (Spicer et al., 2021; Wang et al., 2021; Zhao et al., 2021). Although previously published data showed that more than 40% of NSCLC patients achieved major pathological response after neoadjuvant immunochemotherapy (NAIC), a large proportion of patients still could not benefit from such treatment modality and developed disease progression rapidly (Jiang et al., 2022). As the clinical management strategies for responders significantly differ from nonresponders, a non-invasive method to early evaluate, or even predict the treatment response to NAIC is urgently needed.

18F-FDG PET/CT, with its ability to depict both anatomical and functional changes, has been widely applied in oncological routines. The glucometabolism within a tumor, as revealed by PET imaging, was found to correlate with the expression of immune checkpoint programmed death 1 (PD-1), and the quantitative measurement of metabolic changes was found to associate with pathological response to NAIC (Lopci et al., 2016; Tao et al., 2020). Hence, 18F-FDG PET/CT might be utilized for early response evaluation. Prior studies have proposed several response-evaluation criteria for novel immunotherapies, of which the PET Response Criteria in Solid Tumors (PERCIST) (Joo Hyun et al., 2016) and the immune PERCIST (iPERCIST) standards were the most well-known (Goldfarb et al., 2019). Despite the reasonable diagnostic and predictive performance, their clinical implementation was hindered by several drawbacks (Tao et al., 2020). Both criteria require SULpeak, a value that reveals the average SUV within a small sphere (usually 1.2 cm in diameter) around the most hypermetabolic voxel, rendering small lesions with a metabolic diameter of less than 1 cm unmeasurable. Moreover, the partial volume effect might incur considerable measuring bias (Soret et al., 2007). Compared with SULpeak, a SULmax value, which reflects the most metabolically active portion of a potentially heterogeneous mass, was applicable in most lesions, thus is routinely reported and more clinically feasible (Wahl et al., 2009; Lodge et al., 2012). Moreover, prior studies found SULmax highly reproducible and comparable with proper standardization of the scanning protocol, despite the theoretical reproducibility issues. Hence, we hypothesize that a SULmax-based protocol might be clinically practical yet robust for the response evaluation and prediction of NAIC.

In light of the above, we proposed a SULmax-based protocol, named iPERCIST-max, to evaluate and predict treatment response to NAIC and tested our model’s diagnostic and prognostic performance in a training and an independent external validation cohort.

## Materials and methods

### Patients and study design

This prospective study was proved by the institutional review board (The Ethics Committee of the Guangdong Provincial People’s Hospital, No. GDREC2019687H). All procedures involved in this study were performed in accordance with the Declaration of Helsinki (as revised in 2013). Written informed consent was obtained from each participant in the study.

Data were collected from two prospectively maintained cohorts starting from May 2019 to December 2021: 1) A training cohort from our local institute and 2) an external validation cohort from an independent institute from Sun Yat-sen University Cancer Center.

The included patients met the following criteria: 1) Pathologically confirmed as NSCLC (Stage IB-IIIC AJCC 8th edition) that was potentially resectable after neoadjuvant therapy; 2) treatment-naïve and adequate organ function. Exclusion criteria were as follows: 1) Epidermal growth factor receptor (EGFR)-sensitive mutation; 2) previous autoimmune disease; 3) prior treatment with drugs that target T-cell co-stimulation pathways (such as checkpoint inhibitors).

**FIGURE 1:**
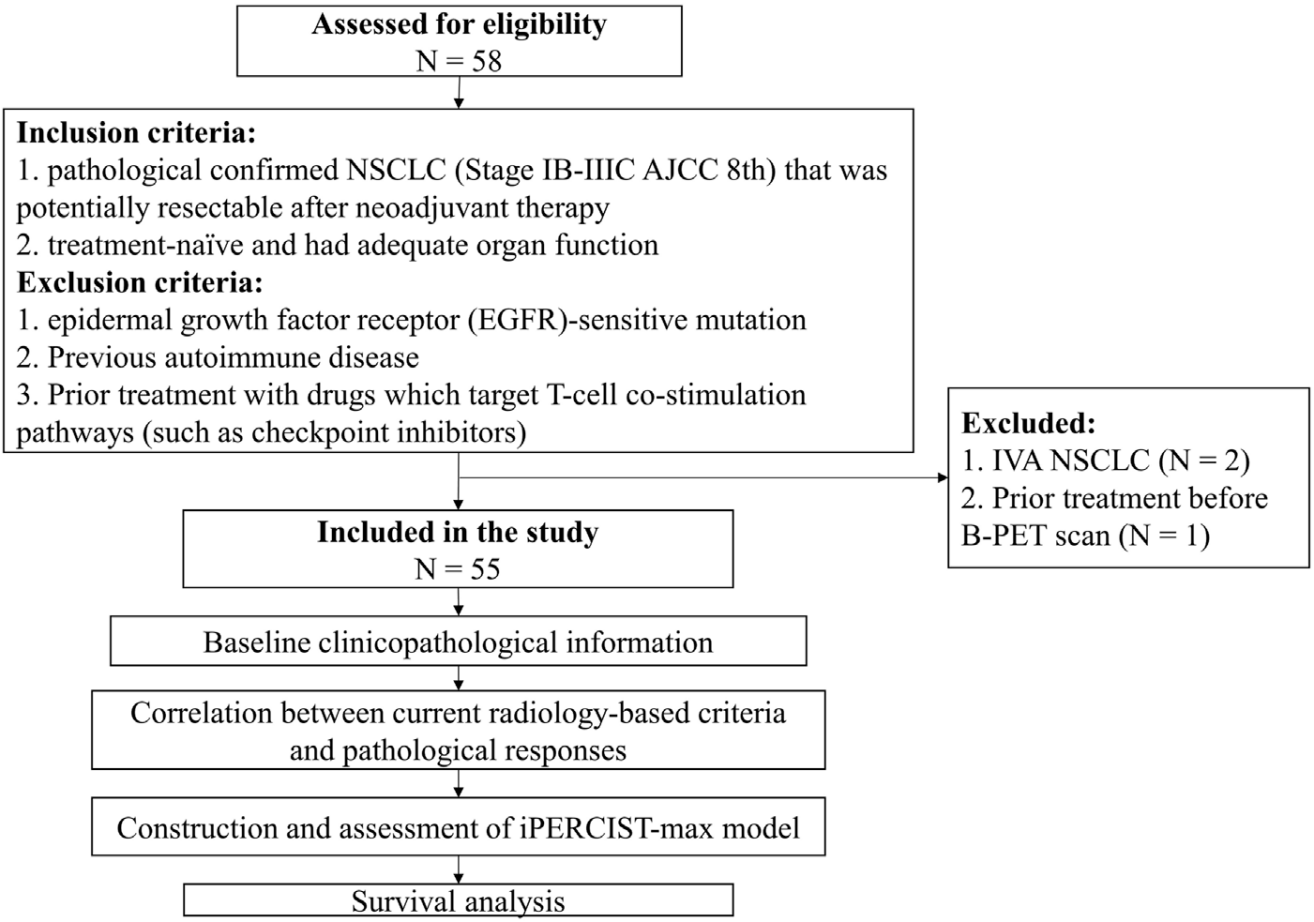
Flowchart of the study design. Fifty-eight patients were assessed for eligibility; eventually, fifty-five cases were included for subsequent analysis.

A flowchart describing the study design is presented in Figure 1. All patients underwent baseline ^18^F-FDG PET/CT (B-PET) examination within 14 days before neoadjuvant therapy incorporating PD-1 inhibitors (200 mg) plus platinum-doublet chemotherapy every 3 weeks. After 2-4 cycles of neoadjuvant therapy, a follow-up ^18^F-FDG PET/CT (F-PET) was performed. When F-PET showed progressive disease, a second F-PET was performed after 4–8 weeks (Goldfarb et al., 2019).

### Image acquisition and analysis

All patients in our training cohort were scanned using the same scanner (Biograph HI-REZ 16; Siemens Healthcare, Henkestr, Germany), following the criteria of the uniform protocols for imaging in clinical trials (UPICT) (Graham et al., 2015). Supplementary File S1 describing the details of the imaging protocol was provided. All images were evaluated using a commercial medical image-processing workstation (uWS-MI, United Imaging Healthcare, Shanghai, China). Two experienced nuclear medicine physicians (TT S & YL J) who were blinded to the patients’ clinical information independently analyzed the PET/CT images, resolving disagreements through consensus. Metabolic parameters including SULmax, SULpeak, SULmean, MTV in mL, and TLG were recorded on a lesion basis. Additionally, thoracic CECT images were reviewed by two board-certified radiologists (H Y & XC Z) who were blinded to the PET/CT study.

### Response evaluation

We proposed the iPERCIST-max criteria based on the proportional changes in the SULmax response (△SULmax%) using the following formula:

$$\frac{Subsequent\;SULmax-baseline\;SULmax}{baseline\;SULmax}\times 100\%.$$ (1)

The patients were divided into the following three groups based on tumor responses assessed by △SULmax%: iPERCIST-max complete metabolic responsive disease (imCMRD), responsive metabolic disease (imRMD), and unresponsive metabolic disease (imUMD). The optimal cutoff values for grouping were determined using a X-tile software (Camp et al., 2004).

Additionally, as references, metabolic and morphological responses were also evaluated using iPERCIST and iRECIST criteria (Goldfarb et al., 2019). Specifically, iPERCIST categorized patients into: Immune complete metabolic response (iCMR), partial metabolic response (iPMR), stable disease (iSMD), unconfirmed progressive metabolic disease PMD (iUPMD), and confirmed PMD (iCPMD); whereas iRECIST (Ayati et al., 2021) classified patients into: immune-complete response (iCR), immune-partial response (iPR), immune-stable disease (iSD), immune-confirmed progressive disease (iCPD), and unconfirmed PD (iUPD). Responsive diseases were defined as either metabolically (iCMR & iPMR per iPERCIST or imCMRD & imRMD per IPERCIST-max as mRD) or morphologically (iCR & iPR, as RD) alterations. Likewise, metabolic and morphological unresponsive disease were diagnosed as mUD (iSMD & iCPMD per iPERCIST or imUMD per iPERCIST-max) and UD (iSD & iCPD), respectively. Notably, upon the diagnosis of iUPMD or iUPD, a second F-PET/CECT study was performed 4–8 weeks later to verify the diagnosis (Eisenhauer et al., 2009; Seymour et al., 2017). (Supplementary Table S1 for a comparison of the iPERCIST, iRECIST, and iPERCIST-max criteria).

### Outcome evaluation

Two experienced pathologists independently performed pathological assessments according to the current standard protocol. Pathological complete response (pCR) was defined as no evidence of residual viable tumor after neoadjuvant treatment. The major pathological response (mPR) was defined as less than 10% residual viable tumor following neoadjuvant treatment. Event-free survival (EFS) was calculated from the date of treatment initiation to the date of the first progression (local recurrence of tumor or distant metastasis) or death from any cause (Zheng et al., 2021). Censored data included those who were lost to follow-up or at the time of the final analysis.

### Statistical analysis

Comparisons of intergroup continuous variables were performed using the Student’s t-test or the Mann-Whitney method whenever suitable. Categorical variables were compared using the chi-squared test or Fisher’s exact test, where suitable. The intraclass correlation coefficient (ICC) was calculated for quantification of the agreement between proportion changes in SULmax (△SULmax%) and in SULpeak (△SULpeak%). Notably, an ICC value between 0.81 and 1.00 suggests an excellent agreement (Zhao et al., 2015). Univariate followed by multivariate binary logistic regressions were used to identify independent predictors of mPR, following a three-step approach (Zhou et al., 2022). The power of prediction models was assessed by the area under the curve (AUC) value acquired from the Time-dependent receiver operating characteristic (ROC) curves. All tests were two-tailed, and *p* < 0.05 denotes statistical significance. All statistical analyses were performed using SPSS v26 software (IBM Corporation, Armonk, NY, United States) and R 4.0.0 (R Core Team 2020).

## Results

### Patient characteristics

Fifty-five patients (males: *n* = 50; females: *n* = 5) with a median age of 66 [Interquartile range (IQR) 58–69] years were enrolled in the training cohort, while 13 patients were enrolled in the external validation cohort. Detailed patient characteristics were tabulated in Table 1.

**TABLE 1:** Patient characteristics. Values are presented as n (%) or median (interquartile range).

Characteristics	Training cohort	Validation cohort	*p* Value
**Sex**			0.265
Male	50 (91)	13 (100)	
Female	5 (9)	0 (0)	
**Age (yrs)**			0.001
Median (IQR)	66 (58–69)	57 (47–59)	
**Smoking history**			0.036
Yes	29 (52.7)	11 (84.6)	
No	26 (47.3)	2 (15.4)	
**Lesion location**			0.370
Right upper lobe	18 (32.7)	5 (38.5)	
Right middle lobe	3 (5.5)	1 (7.7)	
Right lower lobe	8 (14.5)	2 (15.4)	
Left upper lobe	20 (36.4)	2 (15.4)	
Left lower lobe	6 (10.9)	3 (23.1)	
**Pathological subtypes**			0.241
Adenocarcinoma	17 (30.9)	4 (30.8)	
Squamous cell carcinoma	35 (63.6)	7 (53.8)	
Others	3 (5.5)	2 (15.4)	
**Clinical stage**			0.345
IB	2 (3.6)	0 (0)	
IIA	5 (9.1)	0 (0)	
IIB	8 (14.5)	1 (7.7)	
IIIA	17 (30.9)	6 (46.2)	
IIIB	19 (34.5)	6 (46.2)	
IIIC	4 (7.3)	0 (0)	
**Pathological stage**			0.650
0	13 (36.1)	8 (66.7)	
IA	9 (25)	0 (0)	
IB	4 (11.1)	1 (8.3)	
IIB	5 (13.9)	1 (8.3)	
IIIA	5 (13.9)	2 (16.7)	
**Lymphovascular invasion**			0.553
Negative	35 (97.2)	12 (100)	
Positive	1 (2.8)	0 (0)	
**Perineural invasion**			1
Negative	36 (100)	12 (100)	
**R0**			0.553
R0	35 (97.2)	12 (100)	
R1	1 (2.8)	0 (0)	

### Treatment responses to NAIC

In the training cohort, 36 patients were deemed surgical candidates after the treatment, and the post-surgical histological studies confirmed that 13 (36.1%) patients achieved pCR while 23 (63.9%) achieved mPR. Likewise, in the independent validation cohort, 12 patients underwent surgery and 9 (75%) patients achieved mPR status.

### PET imaging parameters in mPR and pCR patients

**FIGURE 2:**
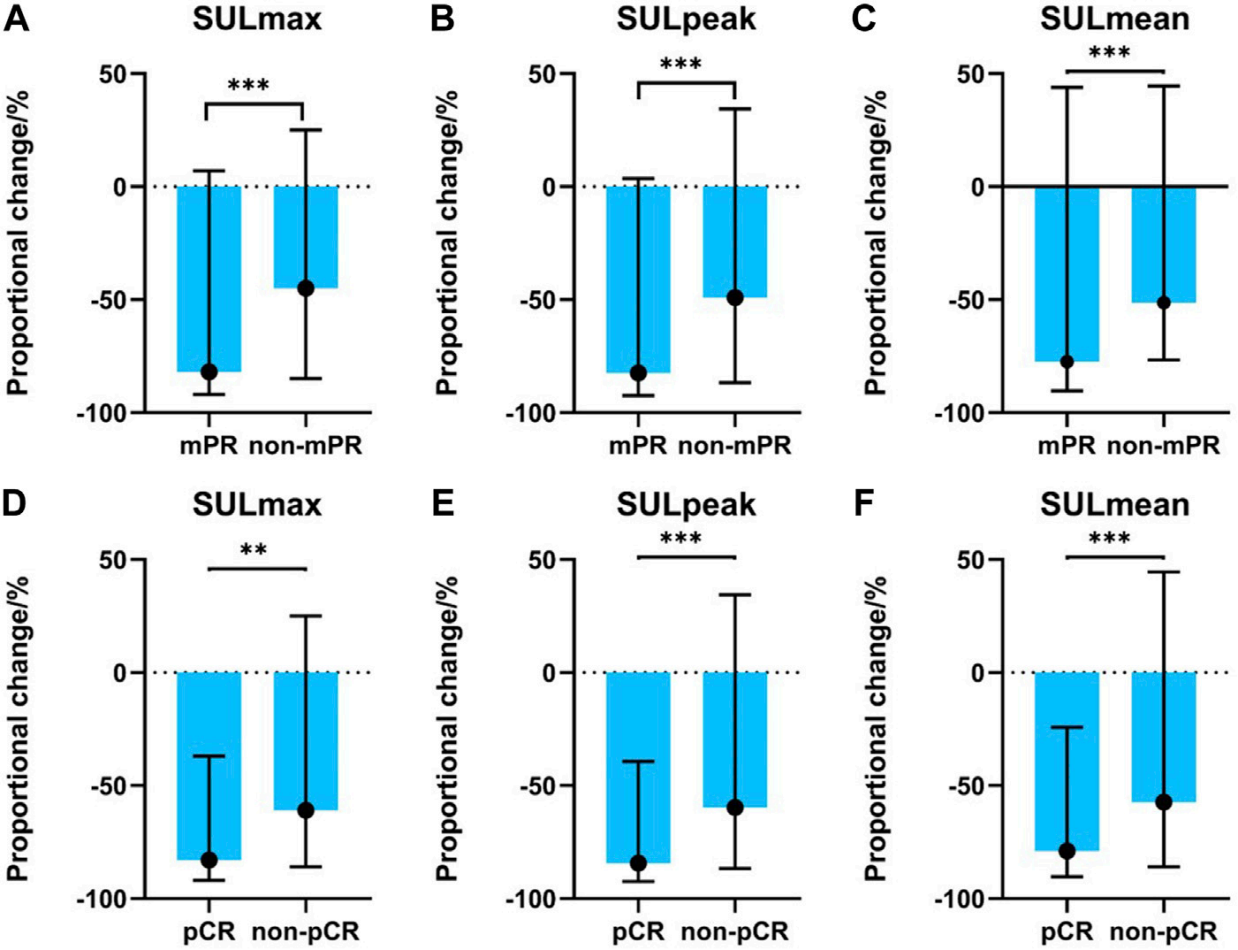
Association between PET-related parameters and pathological responses. **(A)** SULmax between mPR and non-mPR cohorts; **(B)** SULpeak between mPR and non-mPR cohorts; **(C)** SULmean between mPR and non-mPR cohorts; **(D)** SULmax between pCR and non-pCR cohorts; **(E)** SULpeak between pCR and non-pCR cohorts; **(F)** SULmean between pCR and non-pCR cohorts.

Significant alterations in post-treatment SULmax (*p* < 0.001), SULpeak (*p* < 0.001), SULmean (*p* < 0.001), MTV (*p* < 0.001), TLG (*p* < 0.001), and tumor size (*p* < 0.001) were observed compared with baseline values (Supplementary Figure S1) for all patients. Specifically, mPR patients presented with significantly lower SULpeak, SULmax, and SULmean than non-mPR (*p* < 0.05, as shown in Figures 2A–C). The median response rates of SULmax, SULpeak, and SULmean were significantly higher in the pCR cohort than in the non-pCR cohort (Figures 2D–F).

### Comparison of iRECIST and iPERCIST evaluation

**FIGURE 3:**
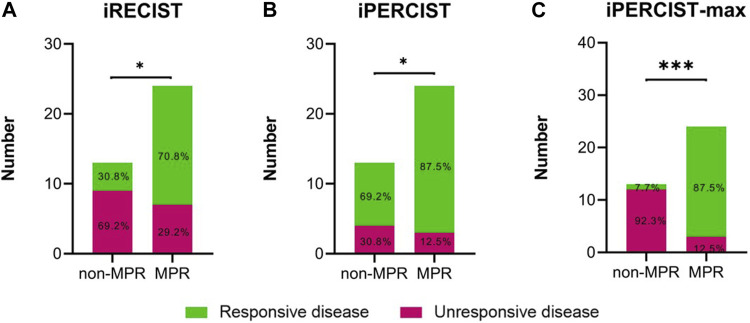
Differences in radiological disease responsiveness between mPR and non-mPR status based on iRECIST/iPERCIST/iPERCIST-max. **(A)** iRECIST; **(B)** iPERCIST; **(C)** iPERCIST-max. Green color represents responsive disease, while red color represents unresponsive disease.

At the first radiological evaluation, 4 of 55 (7.3%) patients were evaluated as iUPD, 20 (36.4%) as iSD, and 31 (56.4%) as iPR. All the iUPD patients were identified as iCPD after the second evaluation. Based on the iRECIST criteria, none of the patients achieved iCR despite 13 pCR cases. Meanwhile, 5 of the 55 (9.1%) patients were classified as iUPMD, seven (12.7%) as iSMD, 38 (69.1%) as iPMR, and five (9.1%) as iCMR based on the iPERCIST criteria. Subsequent PET-scan verified that four out of the five iUPMD cases were iCPMD, and one patient presented with pseudoprogressive disease. Three of the five iCMR patients underwent surgery, and all achieved pCR status. Despite the above stated differences, a significant association was observed in mPR status vs. iRECIST responsiveness (Figure 3A, *p* = 0.019) and iPERCIST responsiveness (Figure 3B, *p* = 0.025). Comparisons in diagnostic indicators among different criteria were shown in Supplementary Table S2.

### Diagnostic performance of the proposed iPERCIST-max method

**FIGURE 4:**
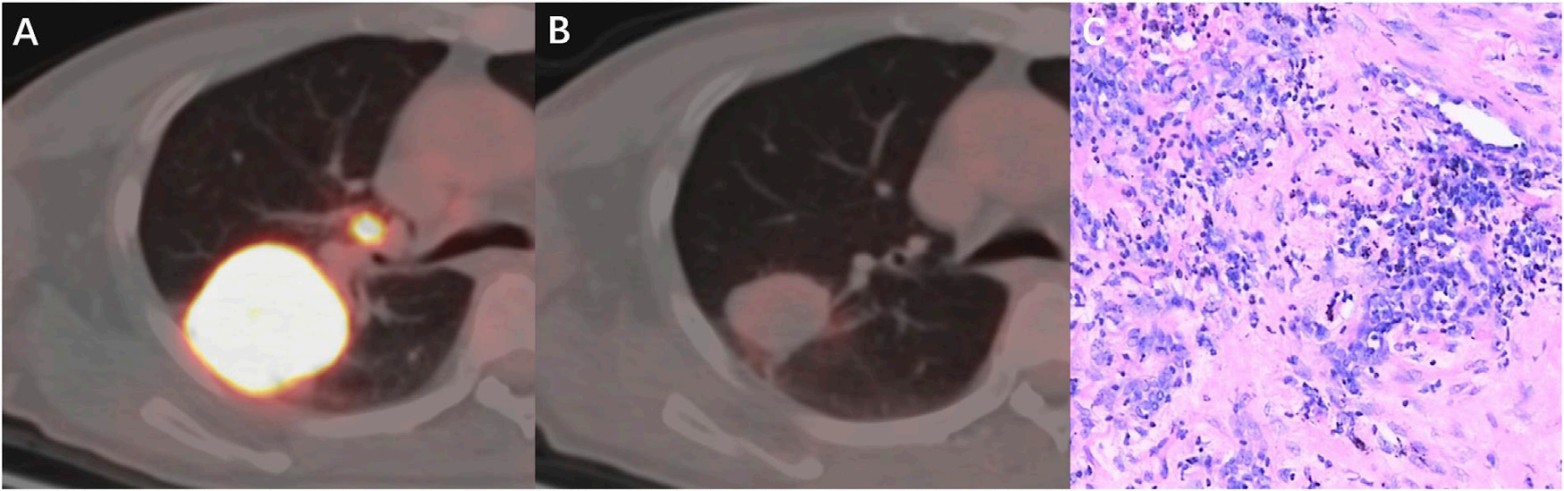
A 53-year-old man with squamous cell carcinoma, who had marked metabolic changes on PET scanning after two cycles of neoadjuvant immunochemotherapy and was classified as responder per iPERCIST-max. **(A)** Baseline PET-CT fusion image of the primary tumor and lymph nodes; **(B)** Follow-up PET-CT fusion image showed markedly reduced metabolic activity of the tumor. **(C)** pathological examination revealed this patient had mPR disease. mPR, major pathological response.

The ICC used to establish the agreement between the SULmax and SULpeak response rates was 0.994 (95% CI 0.990-0.997). The optimal cutoff values of the SULmax response rate were -70.0% and -88.0% using the X-tile software. Subsequently, tumor response represented by △SULmax% higher than -70% was defined as imUMD, those whose SULmax response rate was lower than -70.0% as imRMD, and those whose SULmax response rate was lower than -88.0% as imCMRD. All imCMRD patients who underwent surgery achieved pCR status. Moreover, a significantly higher proportion of imRMD was observed in the mPR cohort than in the non-mPR cohort (χ2 = 12.17, *p* < 0.001) (Figure 3C). Figure 4 showed a typical case who achieved imRMD after two cycles of NAIC and had mPR status after surgery. Furthermore, the univariate and multivariate binary logistic regression showed that iPERCIST-max was the only significant key predictor for mPR status [OR = 84.0, 95% confidence interval (CI): 7.84–900.12, *p* < 0.001] (Table 2).

**TABLE 2:** Univariate and multivariate binary logistic regression analyses. RD: responsive disease; UD: unresponsive disease; MRD: metabolic responsive disease; MUD: metabolic unresponsive disease; cT: clinical T stage; cN: clinical N stage; cTNM: clinical Tumor-nodal-metastasis stage.

Variables	Univariate		Multivariate	
	Odds ratio (95% CI)	*p* value	Odds ratio (95% CI)	*p* value
**Sex**	0.5 (0.062–4.04)	0.516	—	
**Age**	1.079 (0.984–1.184)	0.104	—	
**cT**	1.106 (0.703–1.741)	0.663	—	
**cN**	1.106 (0.703–1.741)	0.663	—	
**cTNM**	1.046 (0.611–1.789)	0.87	—	
**Histological**		—	—	
Adenocarcinoma (ref)	—	—	—	
Squamous cell carcinoma	1.143 (0.257–5.087)	0.861	—	
Others	0.571 (0.028–11.849)	0.718	—	
**Smoking status**	1.379 (0.356–5.341)	0.642	—	
**iRECIST (RD vs. UD)**	5.464 (1.256–23.774)	0.024	—	
**iPERCIST (RD vs. UD)**	3.111 (0.575–16.833)	0.188	—	
**iPERCIST max (MRD vs. MUD)**	73.333 (6.789–792.167)	<0.001	84 (7.839–900.116)	<0.001

We utilized ROC and AUC values to better quantify the overall discriminatory power of various prediction models in predicting the mPR status. ROC analyses for comparisons in mPR status among iRECIST, iPERCIST, and iPERCIST-max were shown in Supplementary Figure S2. The AUC values for iRECIST responsiveness, iPERCIST responsiveness, and iPERCIST-max were 0.688 (95% CI: 0.498–0.877, *p* = 0.070), 0.625 (95% CI: 0.419–0.831, *p* = 0.227), and 0.896 (95% CI: 0.776–1.000, *p* < 0.001), respectively.

Furthermore, the Sun Yat-Sen University Cancer Center (SYSUCC) cohort was used to externally validate the robustness of iPERCIST-max. AUC value for iPERCIST-max predicting mPR status was 0.889 (95% CI: 0.698–1.000, *p* = 0.05).

### The relationship between the iPERCIST-max responsiveness and event-free survival

**FIGURE 5:**
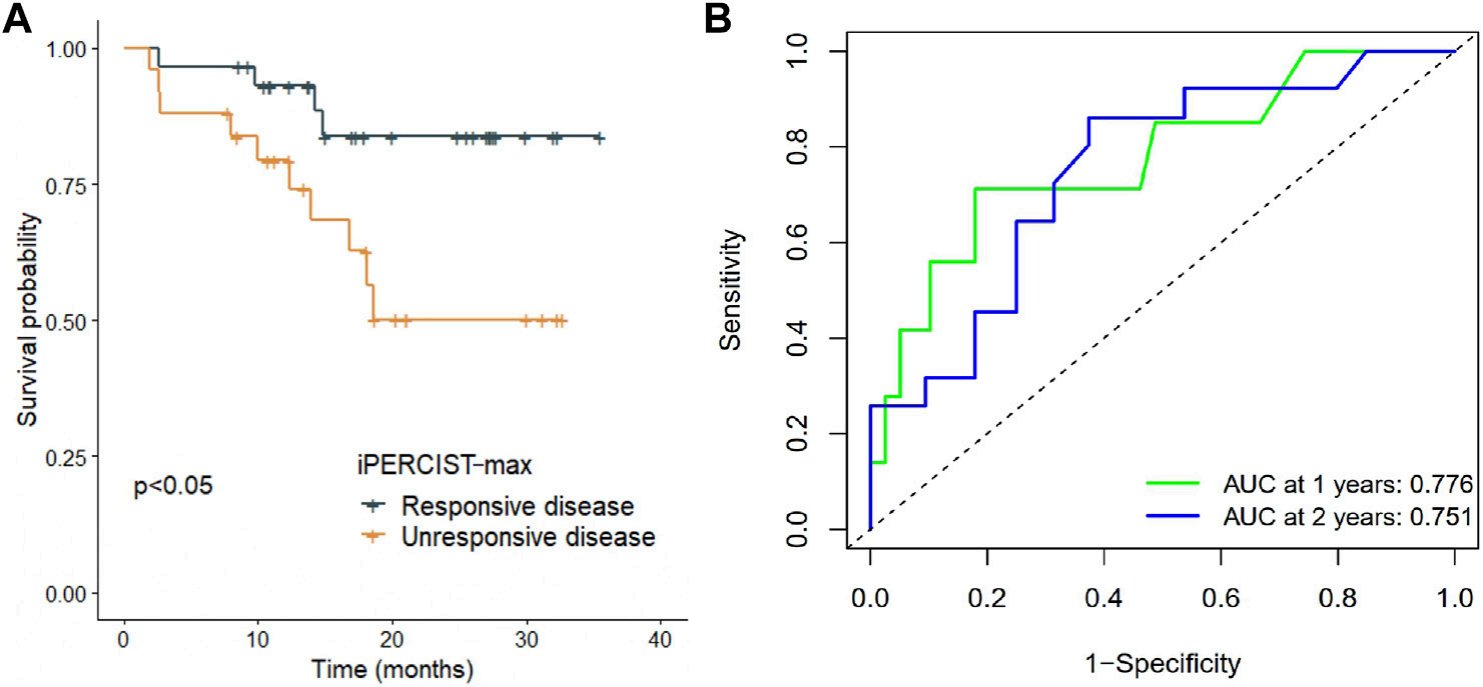
Survival difference in iPERCIST-max groups and internal validation of the robustness of iPERCSIT-max. **(A)** Kaplan-Meier estimated event-free survival between the responsive disease group and unresponsive disease group stratified by iPERCIST-max. **(B)** Time-dependent receiver-operating-characteristics (ROC) analysis for assessment of iPERCIST-max.

The longest and median follow-up time was 35.5 and 21.0 months, respectively. iRECIST, iPERCIST, and iPERCIST-max can stratify patients into distinctive survival groups. Interestingly, no events (recurrence, metastasis, death, etc.) occurred in the imCMRD group at the last follow-up. The imCMRD group exhibited superior EFS compared to the other groups classified by iPERCIST-max (*p* < 0.001). Significantly better EFS in iPERCIST-max mRD (31.5 months, 95% CI: 27.9–35.1) than that in iPERCIST-max mUD (22.2 months, 95% CI: 17.3–27.1 months, *p* = 0.024) was observed (Figure 5A). Moreover, the 1-year survival AUC was 0.776 (95% CI: 0.575–0.976; *p* < 0.001) (Figure 5B). Significant differences in EFS were also seen between the UD and RD cohorts when classified using the iRECIST responsiveness (*p* = 0.033) and iPERCIST responsiveness (*p* = 0.004).

## Discussion

Although the NSCLC treatment is profoundly shifting in the treatment paradigm due to recent advances in immunotherapy (Spicer et al., 2021; Zhao et al., 2021), it is clinically challenged by the lack of a robust and easy-to-use method to distinguish responders from non-responders**.** Hence, we proposed an iPERCIST-max model to predict the treatment response to NAIC. We found this method non-inferior to iPERCIST and iRECIST criteria in terms of robustness (Figure 3) but outperformed the latter in feasibility due to more straightforward clinical applications. Thus, the iPERCIST-max model might better suit the response evaluation to NAIC in NSCLC patients than iPERCIST and iRECIST criteria.

iPERCIST has been validated in multiple studies for its performance in assessing treatment responses to immunotherapies, using proportional changes in SULpeak values (Tao et al., 2020). However, as shown in our research, its clinical application was significantly challenged by its inability to index small lesions, mainly nodal and pulmonary metastasis [21 of 132 (15.6%) at baseline and 56 of 132 (42.2%) at follow-up]. Interestingly, SULmax, which was believed to be affected by multiple factors such as quanton noises, thus lacks the reproducibility for a quantitative study, was found effective in the treatment evaluation in this study. We hypothesize that with the modern advancement of novel PET hardware and proper standardization of the imaging acquisition protocol, the variation of SULmax has been minimized, evidenced by a high agreement between the SULpeak and SULmax measurement in this study [ICC = 0.994 (95% CI 0.990-0.997)] and in others (Kumar et al., 2013).

Owing to the broader clinical applicability of SULmax, we decided to develop iPERCIST-max based on △SULmax%. Using −70.0% as the cutoff value calculated from the X-tile, the iPERCIST-max divided patients into imCMRD, imRMD and imUMD.

Subsequent binary logistic regression and ROC curve analysis demonstrated the ability of iPERCIST-max to predict the mPR status. Furthermore, external validation using an independent SYSUCC cohort further enhanced the applicability of iPERCIST-max.

The difference in the mPR prediction power between iPERCIST-max and iPERCIST is mainly due to the choice of the optimal cutoff value. The cutoff value for the iPERCIST responsiveness-based model was 30%, the selection of which could falsely include patients with non-mPR status. Tao et al. reported that an increase in △SULmax% and △SULpeak% was positively associated with the degree of pathological regression, which indicated that pathological responses that achieved mPR were significantly associated with a −90% proportional change in metabolism. From this aspect, it is reasonable to locate a cut-off value higher than 30%.

Beer et al. and Ayati et al. reported better overall survival (OS) and progression-free survival in the RD cohort than in the UD cohort classified by the PERCIST and RECIST cohorts (Beer et al., 2019; Ayati et al., 2021). Similarly, our study revealed that both CT-based and PET-based criteria could stratify the survival difference between radiological/metabolic responders and nonresponders. In contrast to these findings, Rossi et al. showed no significant difference in OS prediction ability between morphological- and metabolic-based criteria (Rossi et al., 2020), indicating that PERCIST or iPERCIST may have limited clinical applicability in certain cohorts. In the present study, further survival analysis showed that the iPERCIST-max model could not only predict mPR status but could also stratify patients into ordinal survival groups (Figure 5A). The AUC value at 1 year and 2 years reached 0.776 (95% CI, 0.575–0.976; *p* < 0.001) and 0.751 (95% CI, 0.637–0.957) respectively, suggesting the robustness of the iPERCIST-max model.

Pseudo-progression might challenge the treatment evaluation, leading to premature termination of a potentially effective treatment; however, our data showed a low incidence of pseudo-progression (1 in 55,1.8%), which is in accordance with published data (Chiou and Burotto, 2015; Katz et al., 2018; Lee et al., 2018).

Despite these promising results, this study has several limitations. The sample size is small, and we excluded patients with EGFR-mutant adenocarcinoma, resulting in a biased disease population, which might prohibit the direct generalization of our conclusion into other cohorts. Further large-scale prospective studies with an unbiased disease population (without exclusion of adenocarcinoma) are needed to validate our proposal.

## Conclusion

iPERCSIT-max based on temporal changes in PET metabolic parameters, particularly △SULmax% could better predict both early pathological tumor response and prognosis of NSCLC treated with NAIC than commonly used criteria.

## Data Availability

The raw data supporting the conclusion of this article will be made available by the authors, without undue reservation.

## Supplementary material

Supplementary Figure S1 Paired sample test for PET-associated metabolic characteristics. **(A)** SULmax. **(B)** SULpeak; **(C)** SULmean; **(D)** MTV; **(E)** TLG; **(F)** size. Blue dots represent patients at baseline PET-CT evaluation, while red dots represents patients at the final time of tumor response evaluation. *, *p* < 0.05; **, *p* < 0.01, ***, *p* < 0.001. MTV, metabolic tumor volume; TLG, total lesion glycolysis; PreT: pretreatment; PostT: posttreatment.

Supplementary Figure S2 Receiver-operating-characteristics (ROC) analysis for assessment of iRECIST, iPERCIST and iPERCIST-max in predicting major pathological response status. **(A)** iRECIST; **(B)** iPERCIST; **(C)** iPERCIST-max.

## Glossary

AUC: area under the curve
B-PET: baseline 18F-FDG PET/CT
EFS: event-free survival
EGFR: epidermal growth factor receptor
F-PET: follow-up PET/CT
ICC: intraclass correlation coefficient
ICIs: Immune checkpoint inhibitors
iPERCIST-max: the immune PET Response Criteria in Solid Tumors *via* SULmax
iPERCIST: the immune PERCIST
imCMRD: iPERCIST-max complete metabolic responsive disease
imRMD: iPERCIST-max responsive metabolic disease
imUMD: iPERCIST-max unresponsive metabolic disease
iCMR: immune complete metabolic response
iPMR: immune partial metabolic response
iSMD: immune stable disease
iUPMD: immune unconfirmed progressive metabolic disease
iCR: immune-complete response
iPR: immune-partial response
iSD: immune-stable disease
i(C/U)PD: immune (confirmed/unconfirmed) progressive disease
mPR: major pathological response
NSCLC: non-small cell lung cancer
MTV: metabolic tumor volume
NAIC: neoadjuvant immunochemotherapy
PD-1: programmed death 1
pCR: pathological complete response
PERCIST: PET Response Criteria in Solid Tumors
SYSUCC: Sun Yat-Sen University Cancer Center
△SULmax%: proportional changes in the SULmax response
TLG: total lesion glycolysis
UPICT: uniform protocols for imaging in clinical trials

## References

[B1] Ayati N. Lee S. T. Zakavi S. R. Cheng M. Lau W. F. E. Parakh S. (2021). Response evaluation and survival prediction after PD-1 immunotherapy in patients with non-small cell lung cancer: Comparison of assessment methods. J. Nucl. Med. 62, 926–933. 10.2967/jnumed.120.254508 33246978

[B2] Beer L. Hochmair M. Haug A. R. Schwabel B. Kifjak D. Wadsak W. (2019). Comparison of RECIST, iRECIST, and PERCIST for the evaluation of response to PD-1/PD-L1 blockade therapy in patients with non-small cell lung cancer. Clin. Nucl. Med. 44, 535–543. 10.1097/rlu.0000000000002603 31021918

[B3] Camp R. L. Dolled-Filhart M. Rimm D. L. (2004). X-Tile. Clin. Cancer Res. 10, 7252–7259. 10.1158/1078-0432.ccr-04-0713 15534099

[B4] Chiou V. L. Burotto M. (2015). Pseudoprogression and immune-related response in solid tumors. J. Clin. Oncol. official J. Am. Soc. Clin. Oncol. 33, 3541–3543. 10.1200/jco.2015.61.6870 PMC4622096 26261262

[B5] Eisenhauer E. A. Therasse P. Bogaerts J. Schwartz L. H. Sargent D. Ford R. (2009). New response evaluation criteria in solid tumours: Revised RECIST guideline (version 1.1). Eur. J. Cancer 45, 228–247. 10.1016/j.ejca.2008.10.026 19097774

[B6] Goldfarb L. Duchemann B. Chouahnia K. Zelek L. Soussan M. (2019). Monitoring anti-PD-1-based immunotherapy in non-small cell lung cancer with FDG PET: Introduction of iPERCIST. EJNMMI Res. 9, 8. 10.1186/s13550-019-0473-1 30694399 PMC6890907

[B7] Graham M. M. Wahl R. L. Hoffman J. M. Yap J. T. Sunderland J. J. Boellaard R. (2015). Summary of the UPICT protocol for 18F-FDG PET/CT imaging in Oncology clinical trials. J. Nucl. Med. 56, 955–961. 10.2967/jnumed.115.158402 25883122 PMC4587663

[B8] Huang S. Gao Z. Qiao G. (2021). Immunochemotherapy as first-line treatment for locally advanced or metastatic squamous non-small cell lung cancers. JAMA Oncol. 7, 1580. 10.1001/jamaoncol.2021.3372 34410312

[B9] Jiang J. Wang Y. Gao Y. Sugimura H. Minervini F. Uchino J. (2022). Neoadjuvant immunotherapy or chemoimmunotherapy in non-small cell lung cancer: A systematic review and meta-analysis. Transl. Lung Cancer Res. 11, 277–294. 10.21037/tlcr-22-75 35280319 PMC8902081

[B10] Katz S. I. Hammer M. Bagley S. J. Aggarwal C. Bauml J. M. Thompson J. C. (2018). Radiologic pseudoprogression during anti-PD-1 therapy for advanced non-small cell lung cancer. J. Thorac. Oncol. 13, 978–986. 10.1016/j.jtho.2018.04.010 29738824

[B11] Kumar V. Nath K. Berman C. G. Kim J. Tanvetyanon T. Chiappori A. A. (2013). Variance of SUVs for FDG-PET/CT is greater in clinical practice than under ideal study settings. Clin. Nucl. Med. 38, 175–182. 10.1097/rlu.0b013e318279ffdf 23354032 PMC3578161

[B12] Lee J. H. Long G. V. Menzies A. M. Lo S. Guminski A. Whitbourne K. (2018). Association between circulating tumor DNA and pseudoprogression in patients with metastatic melanoma treated with anti-programmed cell death 1 antibodies. JAMA Oncol. 4, 717–721. 10.1001/jamaoncol.2017.5332 29423503 PMC5885201

[B13] Lodge M. A. Chaudhry M. A. Wahl R. L. (2012). Noise considerations for PET quantification using maximum and peak standardized uptake value. J. Nucl. Med. 53, 1041–1047. 10.2967/jnumed.111.101733 22627001 PMC3417317

[B14] Lopci E. Toschi L. Grizzi F. Rahal D. Olivari L. Castino G. F. (2016). Correlation of metabolic information on FDG-PET with tissue expression of immune markers in patients with non-small cell lung cancer (NSCLC) who are candidates for upfront surgery. Eur. J. Nucl. Med. Mol. Imaging 43, 1954–1961. 10.1007/s00259-016-3425-2 27251642

[B15] Joo Hyun O. Lodge M. A. Wahl R. L. (2016). Practical PERCIST: A simplified Guide to PET response criteria in solid tumors 1.0. Radiology 280, 576–584. 10.1148/radiol.2016142043 26909647 PMC4976461

[B16] Ramos-Esquivel A. Van Der Laat A. Rojas-Vigott R. Juárez M. Corrales-Rodríguez L. (2017). Anti-PD-1/anti-PD-L1 immunotherapy versus docetaxel for previously treated advanced non-small cell lung cancer: A systematic review and meta-analysis of randomised clinical trials. ESMO Open 2, e000236. 10.1136/esmoopen-2017-000236 29181191 PMC5699523

[B17] Rossi G. Bauckneht M. Genova C. Rijavec E. Biello F. Mennella S. (2020). Comparison between (18)F-FDG PET-based and CT-based criteria in non-small cell lung cancer patients treated with nivolumab. J. Nucl. Med. 61, 990–998. 10.2967/jnumed.119.233056 31806768

[B18] Seymour L. Bogaerts J. Perrone A. Ford R. Schwartz L. H. Mandrekar S. (2017). iRECIST: guidelines for response criteria for use in trials testing immunotherapeutics. Lancet Oncol. 18, e143–e152. 10.1016/s1470-2045(17)30074-8 28271869 PMC5648544

[B19] Siegel R. L. Miller K. D. Fuchs H. E. Jemal A. (2021). Cancer statistics, 2021. Ca. A Cancer J. Clin. 71, 7–33. 10.3322/caac.21654 33433946

[B20] Soret M. Bacharach S. L. Buvat I. (2007). Partial-volume effect in PET tumor imaging. J. Nucl. Med. 48, 932–945. 10.2967/jnumed.106.035774 17504879

[B21] Spicer J. Wang C. Tanaka F. Saylors G. B. Chen K.-N. Liberman M. (2021). Surgical outcomes from the phase 3 CheckMate 816 trial: Nivolumab (NIVO) + platinum-doublet chemotherapy (chemo) vs chemo alone as neoadjuvant treatment for patients with resectable non-small cell lung cancer (NSCLC). J. Clin. Oncol. 39, 8503. 10.1200/jco.2021.39.15_suppl.8503

[B22] Tao X. Li N. Wu N. He J. Ying J. Gao S. (2020). The efficiency of (18)F-FDG PET-CT for predicting the major pathologic response to the neoadjuvant PD-1 blockade in resectable non-small cell lung cancer. Eur. J. Nucl. Med. Mol. Imaging 47, 1209–1219. 10.1007/s00259-020-04711-3 32043180 PMC7101299

[B23] Uprety D. Mandrekar S. J. Wigle D. Roden A. C. Adjei A. A. (2020). Neoadjuvant immunotherapy for NSCLC: Current concepts and future approaches. J. Thorac. Oncol. 15, 1281–1297. 10.1016/j.jtho.2020.05.020 32522713

[B24] Wahl R. L. Jacene H. Kasamon Y. Lodge M. A. (2009). From RECIST to PERCIST: Evolving Considerations for PET response criteria in solid tumors. J. Nucl. Med. 50 (1), 122s–150s. 10.2967/jnumed.108.057307 19403881 PMC2755245

[B25] Wang J. Lu S. Yu X. Hu Y. Sun Y. Wang Z. (2021). Tislelizumab plus chemotherapy vs chemotherapy alone as first-line treatment for advanced squamous non-small-cell lung cancer: A phase 3 randomized clinical trial. JAMA Oncol. 7, 709–717. 10.1001/jamaoncol.2021.0366 33792623 PMC8017481

[B26] Zhao H. Wang J. Liu X. Zhao X. Hippe D. S. Cao Y. (2015). Assessment of carotid artery atherosclerotic disease by using three-dimensional fast black-blood MR imaging: Comparison with DSA. Radiology 274, 508–516. 10.1148/radiol.14132687 25286322 PMC4314290

[B27] Zhao Z. R. Yang C. P. Chen S. Yu H. Lin Y. B. Lin Y. B. (2021). Phase 2 trial of neoadjuvant toripalimab with chemotherapy for resectable stage III non-small-cell lung cancer. Oncoimmunology 10, 1996000. 10.1080/2162402x.2021.1996000 34712513 PMC8547836

[B28] Zheng Y. Liu X.-B. Sun H.-B. Xu J. Shen S. Ba Y.-F. (2021). Written on Henan Cancer Hospital Thoracic Oncology, GA phase III study on neoadjuvant chemotherapy versus neoadjuvant toripalimab plus chemotherapy for locally advanced esophageal squamous cell carcinoma: Henan Cancer Hospital Thoracic Oncology Group 1909 (HCHTOG1909). Ann. Transl. Med. 9, 73. 10.21037/atm-20-5404 33553366 PMC7859818

[B29] Zhou Z. Huang S. Ben X. Zhuang W. Hong L. Xie Z. (2022). A novel prognostic model: Which group of esophageal squamous cell carcinoma patients could benefit from adjuvant chemotherapy. Ann. Transl. Med. 10, 182. 10.21037/atm-22-46 35280404 PMC8908144

