# Supplementary material for: Dynamic alteration in SULmax predicts early pathological tumor response and short-term prognosis in non-small cell lung cancer treated with neoadjuvant immunochemotherapy
Source: Front Bioeng Biotechnol. 2022 Oct 6;10:1010672. doi: 10.3389/fbioe.2022.1010672 (PMC9582780; doi:10.3389/fbioe.2022.1010672)
Supplement: Supplementary file 1 [file Table1.DOCX]

**Supplementary Methods:**

**Image Acquisition Protocol**

All the procedures involved in PET/CT examination met the Uniform Protocols for Imaging in Clinical Trials (UPICT) 18F-FDG PET/CT protocol [1]. All patients fasted for at least 4 hours before ^18^F-FDG administration. Patients with a blood glucose level higher than 144 mg/dL (8.0 mmol/L) or lower than 72 mg/dL (4.0 mmol/L) were rescheduled. Patients were implanted with 20G indwelling intravenous catheters (Jierui Medical Product), followed by ^18^F-FDG manual administration with 5.92 MBq (0.16 mCi per body weight). PET/CT studies were acquired from mid-thigh to the base of the skull[2] using a total-body PET/CT scanner (Biograph HI-REZ 16, Siemens Healthcare, Henkestr, Germany) after 60 ± 5 min resting in dark room[3]. Subjects were instructed to lie supine on the scanning bed with the arms at their sides during scanning. Firstly, Low dose CT scans (tube current 50 mAs, voltage 120 kV, rotation time 0.5 s, pitch 1.35, collimation 16 × 0.75 mm) of the torso were obtained which reconstructed in a 512 × 512 matrix for attenuation correction and anatomic registration. Emission data were acquired with 2 min/bed position in 3 dimensional mode; and PET images were reconstructed using ordered subset expectation maximization (OSEM) , 4 iterations, 8 subsets, matrix 128 × 128, display field of view (DFOV) of 68.3 cm. Images were reconstructed with a voxel size of []×[]×[] mm^3^ and were smoothed using a post-reconstruction Gaussian filter with a FWHM of 5 mm.

Subsequently, an inhale breath hold thoracic contrast-enhanced CT (CECT) (120kV, 140mAs, rotation time 0.5 s, pitch 1.35, collimation 16 × 0.85 mm) was implemented with a non-ioniciodinated contrast agent (Iopamiro, 370 mg of iodine/ml, Bracco Sine Pharmaceutical Corp. Ltd., Shanghai, China) using the same PET/CT scanner. The thoracic CECT was started 40 s after the intravenous bolus injection of the contrast agentof 1.5ml/kgat 3 ml/sand fushing with 20 mL saline solution at 3 ml/s to the arm through a contrast media injector (Ulrich REF XD 2060, Ulrich Medical, Ulm, Germany)[4]. Patients who had renal insufficiency or a history of iodinated contrast agent allergy were excluded from CECT.The thoracic CECT images were reconstructed using a standard kernel. atient preparation before the examination, PET and contrast-enhanced computed tomography (CECT) image acquisition, and reconstruction between baseline and follow-up evaluations are presented below.

| **Quality control metrics** | **Baseline** | **Evaluation** | **Value** |
| --- | --- | --- | --- |
| Blood glucose (mmol/L) | 5.63 ± 1.02 | 5.68 ± 0.97 | 0.781 |
| FDG Dose (MBq) | 363.00 ± 61.61 | 363.07 ± 68.47 | 0.984 |
| Uptake Time (minute) | 62.05 ± 4.74 | 62.85 ± 4.30 | 0.356 |
| Liver SULmean | 1.92 ± 0.36 | 1.95 ± 0.35 | 0.354 |

**Table.** Difference in quality control metrics between baseline and evaluation timepoint.

All images evaluation hasbeen performed in a commercial medical image processing workstation (uWS-MI, United Imaging Healthcare, Shanghai, China) and reviewed in standard planes. Two nuclear physicians with 12 years of FDG-PET/CTdiagnostic experienceand subspecialized in thoracic malignancies receiving standard iPERCIST training independently analyzed the PET/CT imagesblinded to the clinical data. Discordance were resolved by consensus. Quantitative parameter values were measured semiautomatically over the volume of interests (VOIs) of primary lung malignancies andmetastatic lymph nodes. Clinicalregional lymph nodes were classified with the International Association for the Study of Lung Cancer (IASLC) Lymph Node Map. Semiquantitative parameters SUL_peak_ (standardized uptake value corrected for lean body mass) were calculated in a 1.2-cm-diameter ROI placed on the hottest spot of each PET study[8]. Suspected baseline lesions were defined as those whose SUL_peak_ greater than the liver background (1.5 × SUL_mean_ + 2 SDs of a 3-cm-diameter spherical ROI in the normal right liver lobe)[2]. For patient with diffuse liver infiltration, 2.0 × descending aorta SUL_mean_ + 2 SDs is used for substitution as minimal metabolically measurable lesion activity. The metabolic tumor volume (MTV) was defined using a research prototype segmentation algorithm (auto-adjustment algorithm version 1.0, United Imaging Healthcare).The total lesion glycolysis (TLG) was calculated as MTV multiplied by the mean SUL (TLG = MTV × SUL_mean_). We recorded the SUL_max_, SUL_mean_, MTV in milliliters, and TLG per lesion. The reduction percentage of each parameter was calculated and recorded.

Thoracic CECT images were reviewed by two board-certified radiologists with more than 10 years of experience blinded to the PET/CT study. Longest diameter of the primary lesion and the short axis of metastatic lymph nodes were recorded [5].

**Reference:**

1. Graham, M.M., et al., *Summary of the UPICT Protocol for 18F-FDG PET/CT Imaging in Oncology Clinical Trials.* J Nucl Med, 2015. **56**(6): p. 955-61.

2. Boellaard, R., et al., *FDG PET/CT: EANM procedure guidelines for tumour imaging: version 2.0.* Eur J Nucl Med Mol Imaging, 2015. **42**(2): p. 328-54.

3. O, J.H., M.A. Lodge, and R.L. Wahl, *Practical PERCIST: A Simplified Guide to PET Response Criteria in Solid Tumors 1.0.* Radiology, 2016. **280**(2): p. 576-84.

4. Wang, S.Y., et al., *PET/CT and contrast-enhanced CT imaging findings in benign solitary schwannomas.* Eur J Radiol, 2021. **141**: p. 109820.

5. Eisenhauer, E.A., et al., *New response evaluation criteria in solid tumours: revised RECIST guideline (version 1.1).* Eur J Cancer, 2009. **45**(2): p. 228-47.
