# Supplementary figures and images for: Dynamic alteration in SULmax predicts early pathological tumor response and short-term prognosis in non-small cell lung cancer treated with neoadjuvant immunochemotherapy
Source: Front Bioeng Biotechnol. 2022 Oct 6;10:1010672. doi: 10.3389/fbioe.2022.1010672 (PMC9582780; doi:10.3389/fbioe.2022.1010672)

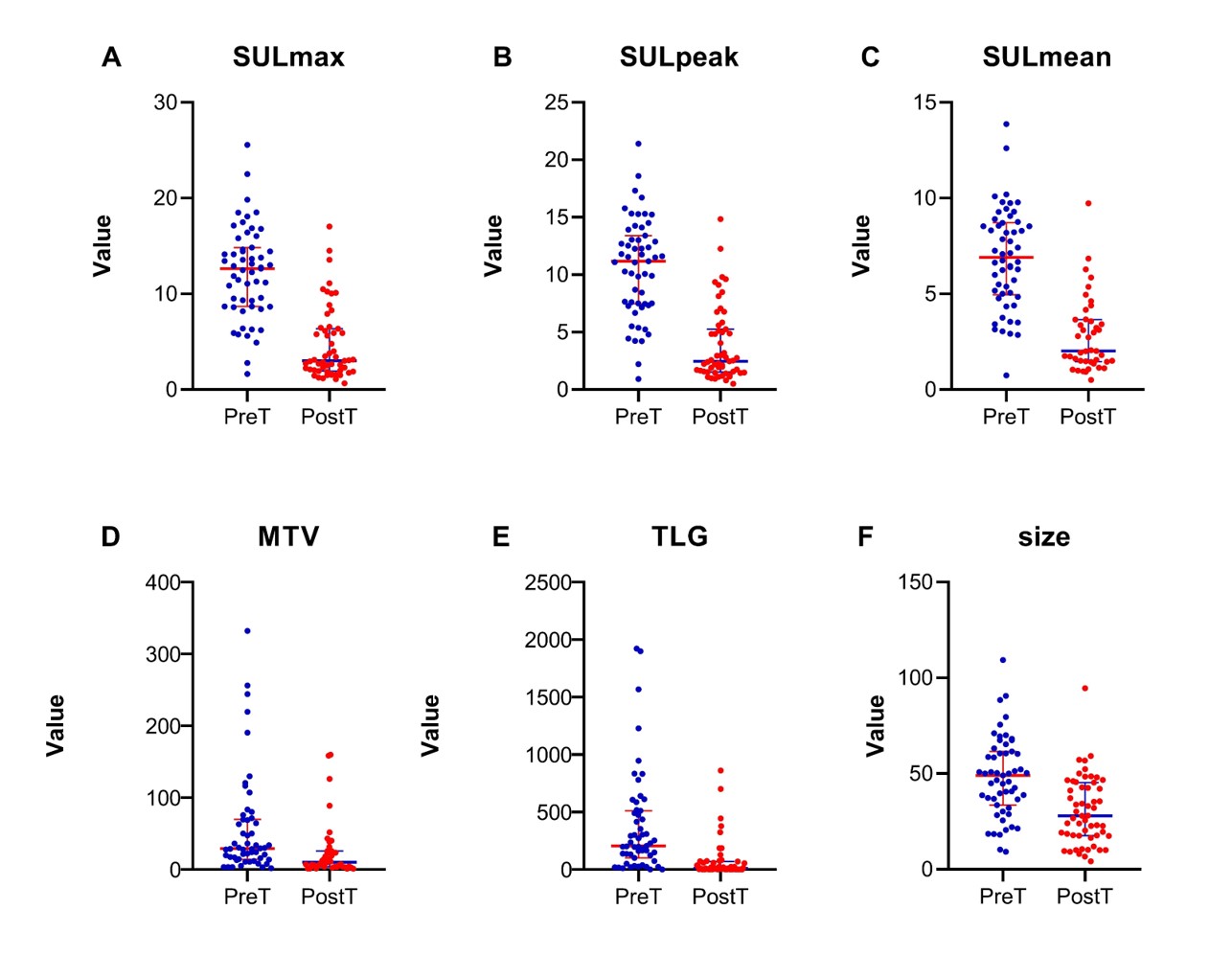

Supplement: Supplementary file 2 [file Image1.JPEG]

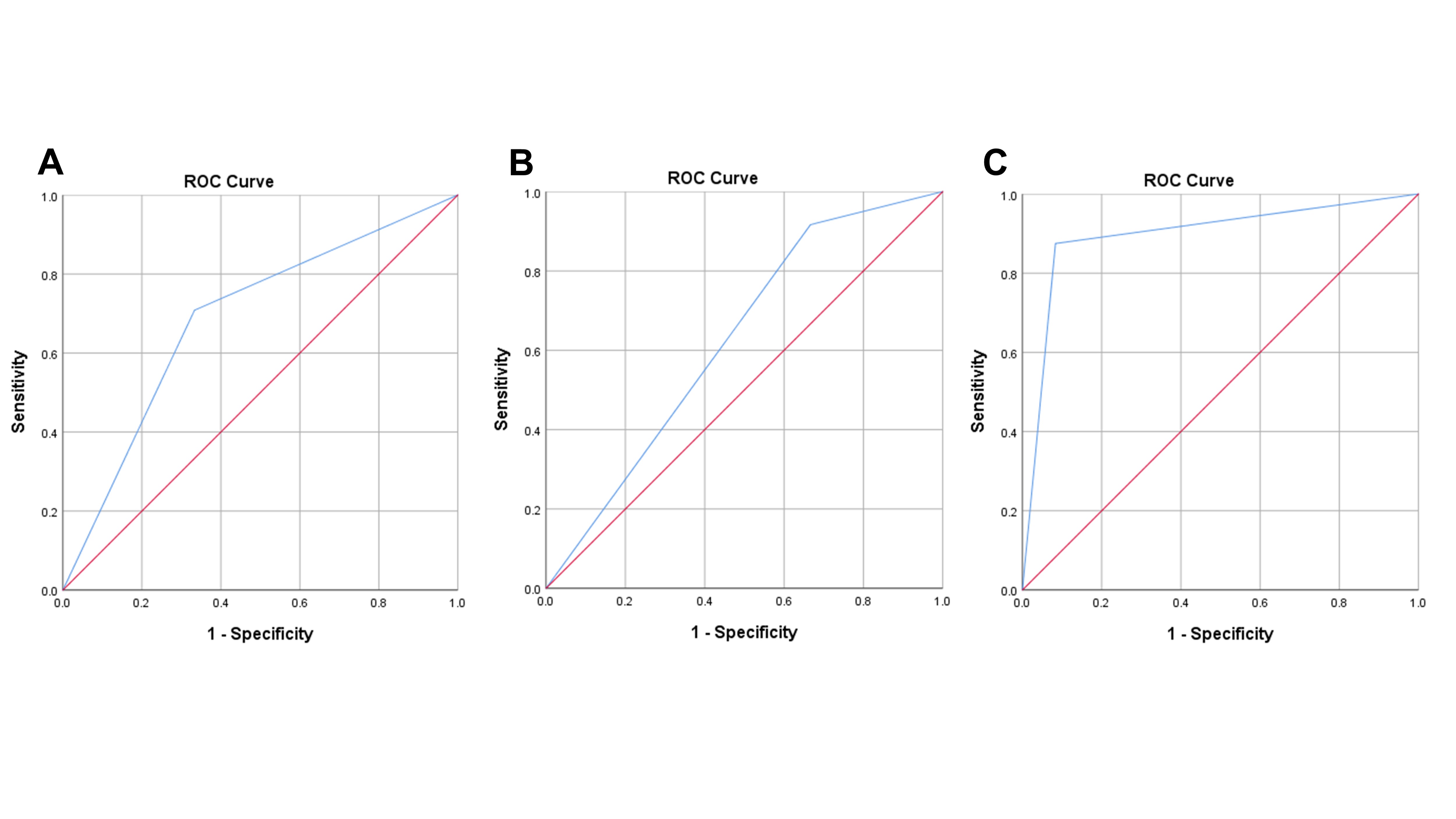

Supplement: Supplementary file 3 [file Image2.JPEG]
