# Supplementary material for: Dynamic alteration in SULmax predicts early pathological tumor response and short-term prognosis in non-small cell lung cancer treated with neoadjuvant immunochemotherapy
Source: Front Bioeng Biotechnol. 2022 Oct 6;10:1010672. doi: 10.3389/fbioe.2022.1010672 (PMC9582780; doi:10.3389/fbioe.2022.1010672)
Supplement: Supplementary file 4 [file Table2.DOCX]

| iPERCIST | | iRECIST | | iPERCIST-max | |
| --- | --- | --- | --- | --- | --- |
| Responses |  | **Responses** |  | **Responses** | **iPERCIST-max** |
| iCMR | disappearance of all metabolically active lesions | iCR | disappearance of all lesions | imCMRD | SULmax reduction ≥ 88.0% in the hottest target lesions |
| iPMR | SULpeak reduction ≥ 30% in the hottest target lesions | iPR | ≥30% decrease from baseline | imRMD | SULmax reduction falls between 70.0% to 88.0% in the hottest target lesions |
| iSMD | neither PMD nor PMR | iSD | Neither PR nor PD | imUMD | SULmax reduction＜70% |
| iPMD | iUPMD: SULpeak increase ≥ 30%, or new FDG-avid lesions. Confirmation of progression recommended minimum 4 weeks after the first iUPMD assessment. iCPMD: increased SULpeak of all lesions, or appearance of another new lesion. | iPD | iUPD: confirmation of progression recommended minimum 4 weeks after the first iUPD assessment. iCPD: increased size of all lesions, or appearance of another new lesion |  |  |
| New Lesions | iUPMD, need to be confirmed after 4–8 weeks (iCPMD) | New Lesions | iUPD, not incorporated into tumor burden; confirmed 4–12 weeks apart (iCPD) |  |  |

**Supplementary Table 1. Comparison among iPERCIST, iRECST and iPERCIST-max criteria** imCMRD: iPERCIST-max complete metabolic responsive disease; imRMD: iPERCIST-max responsive metabolic disease; imUMD: iPERCIST-max unresponsive metabolic disease; iCMR: immune complete metabolic response; iPMR: immune partial metabolic response; iSMD: immune stable disease; iUPMD: immune unconfirmed progressive metabolic disease; iCR: immune-complete response, iPR: immune-partial response, iSD: immune-stable disease, i(C/U)PD: immune (confirmed/unconfirmed) progressive disease
