# Supplementary material for: Dynamic alteration in SULmax predicts early pathological tumor response and short-term prognosis in non-small cell lung cancer treated with neoadjuvant immunochemotherapy
Source: Front Bioeng Biotechnol. 2022 Oct 6;10:1010672. doi: 10.3389/fbioe.2022.1010672 (PMC9582780; doi:10.3389/fbioe.2022.1010672)
Supplement: Supplementary file 5 [file Table3.DOCX]

| **Statistic** | **Value (95% CI)** | | |
| --- | --- | --- | --- |
|  | **iRECIST** | **iPERCIST** | **iPERCIST-max** |
| Sensitivity | 70.83% (48.91% - 87.38%) | 87.5% (67.64% - 97.34%) | 87.5% (67.64% - 97.34%) |
| Specificity | 69.23% (38.57% - 90.91%) | 30.77% (9.09% - 61.43%) | 92.31% (63.97% - 99.81%) |
| Positive Likelihood Ratio | 2.3 (0.98 - 5.41) | 1.26 (0.85 - 1.87) | 11.38 (1.72 - 75.23) |
| Negative Likelihood Ratio | 0.42 (0.20 - 0.87) | 0.41 (0.11 - 1.55) | 0.14 (0.05 - 0.39) |
| Disease prevalence | 64.86% (47.46% - 79.79%) | 64.86% (47.46% - 79.79%) | 64.86% (47.46% - 79.79%) |
| Positive Predictive Value | 80.95% (64.38% - 90.90%) | 70% (61.17% - 77.56%) | 95.45% (76.05% - 99.29%) |
| Negative Predictive Value | 56.25% (38.47% - 72.56%) | 57.14% (25.95% - 83.53%) | 80% (57.84% - 92.10%) |
| Accuracy | 70.27% (53.02% - 84.13%) | 67.57% (50.21% - 81.99%) | 89.19% (74.58% - 96.97%) |

**Supplementary Table 2. Comparison in diagnostic indicators among different criteria.**
